# Regional differences in self-reported screening, prevalence and management of cardiovascular risk factors in Switzerland

**DOI:** 10.1186/1471-2458-12-246

**Published:** 2012-03-28

**Authors:** Pedro Marques-Vidal, Fred Paccaud

**Affiliations:** 1 Institute of Social and Preventive Medicine (IUMSP), Route de la Corniche 2, 1066 Epalinges, Switzerland

**Keywords:** Geographical differences, Diabetes, Dyslipidemia, Hypertension, Smoking, Obesity, Switzerland

## Abstract

**Background:**

In Switzerland, health policies are decided at the local level, but little is known regarding their impact on the screening and management of cardiovascular risk factors (CVRFs). We thus aimed at assessing geographical levels of CVRFs in Switzerland.

**Methods:**

Swiss Health Survey for 2007 (N = 17,879). Seven administrative regions were defined: West (Leman), West-Central (Mittelland), Zurich, South (Ticino), North-West, East and Central Switzerland. Obesity, smoking, hypertension, dyslipidemia and diabetes prevalence, treatment and screening within the last 12 months were assessed by interview.

**Results:**

After multivariate adjustment for age, gender, educational level, marital status and Swiss citizenship, no significant differences were found between regions regarding prevalence of obesity or current smoking. Similarly, no differences were found regarding hypertension screening and prevalence. Two thirds of subjects who had been told they had high blood pressure were treated, the lowest treatment rates being found in East Switzerland: odds-ratio and [95% confidence interval] 0.65 [0.50-0.85]. Screening for hypercholesterolemia was more frequently reported in French (Leman) and Italian (Ticino) speaking regions. Four out of ten participants who had been told they had high cholesterol levels were treated and the lowest treatment rates were found in German-speaking regions. Screening for diabetes was higher in Ticino (1.24 [1.09 - 1.42]). Six out of ten participants who had been told they had diabetes were treated, the lowest treatment rates were found for German-speaking regions.

**Conclusions:**

In Switzerland, cardiovascular risk factor screening and management differ between regions and these differences cannot be accounted for by differences in populations' characteristics. Management of most cardiovascular risk factors could be improved.

## Background

Coronary heart disease is one of the leading causes of death worldwide and shows considerable geographical variation [1,2]. This geographical variation can either be due to a true difference in cardiovascular risk factor levels [3-5] or to differences in cardiovascular risk factor management [6-9], although other causes have been suggested [10].

In Switzerland, health policies are decided at the local (canton) level. Further, with four official languages, Switzerland is a country of large socio- cultural diversity, which might also be reflected in health policies and health expenditures [11]. Indeed, smoking policies differ between cantons [12], and significant regional differences in glycemic control have also been reported [13,14]. Hence, it is likely that these differing health policies might lead to differences in cardiovascular risk factor (CVRF) screening and management.

In this study, we used the data from the Swiss Health Survey 2007 to assess the prevalence and management of CVRFs according to geographical region.

## Methods

### Sampling

Data for the Swiss Health Survey (SHS) 2007 were obtained from the Swiss federal bureau of statistics http://www.bfs.admin.ch. The SHS is a cross-sectional, nationwide, population-based telephone survey conducted every 5 years since 1992 by the Federal Statistical Office of Switzerland under a mandate from the federal government [15]. The data can be obtained upon request and for selected purposes.

**Figure 1:**
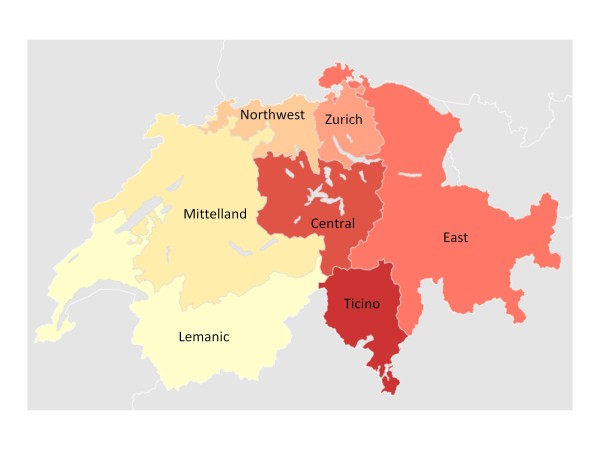
**The seven administrative areas of Switzerland**.

The study population was chosen by stratified random sampling of a database of all private Swiss households with fixed-line telephones (as opposed to mobile phones). Switzerland has one of the highest coverage of fixed phone lines in the world^1 ^and over 90% of the Swiss households have fixed telephones. The first sampling stratum consisted of the seven main regions: West "Leman", West-Central "Mittelland", Northwest, Zurich, North-Eastern, Central and South (Ticino), see Figure 1. The second stratum consisted of the cantons, and the number of households drawn was proportional to the population of the canton. In some cantons, oversampling of households was performed to obtain accurate cantonal estimates. The third stratum consisted of the household. One member of the household was randomly selected in advance within all members aged 15 years and over. A letter inviting this selected household member to participate in the survey was sent to each sampled subject, who was contacted thereafter by phone and interviewed using computer-assisted telephone interview software to manage dialling and data collection. Face-to-face interviews were organised for subjects older than 75 years. In the case of long-term absence of a sampled subject, a proxy interviewee was requested to provide answers on behalf of the pre-defined sampled person (approximately 3% of households). The interviews were carried out in German, French or Italian, as appropriate. People who did not speak any of these three languages were excluded from the survey. Other criteria for exclusion were asylum seeker status, households without a fixed-line telephone, very poor health status and living in a nursing home [16]. The participation rate was 66%. It is estimated that < 2% of households were excluded owing to these exclusion criteria. Details are available at http://www.bfs.admin.ch/bfs/portal/fr/index/infothek/erhebungen__quellen/blank/blank/ess/01.html. In this study, only adult (≥18 years) participants were considered.

### Cardiovascular risk factors screening, prevalence and management

Screening, prevalence and management of the main cardiovascular risk factors was obtained by questionnaire and was thus self-reported. Body mass index (BMI) was computed from self-reported height and weight; overweight was considered for a BMI ≥25 and < 30 kg/m^2^; obesity was considered for a BMI ≥30 kg/m^2^. Smoking status was considered as current, former and never. Current smokers were also asked if they had ever tried to stop smoking for at least two weeks within the last 12 months, but were still considered as smokers in the statistical analysis.

Screening for hypertension, hypercholesterolemia or diabetes was present if the participant reported being screened for this condition within the last 12 months. The prevalence of hypertension, hypercholesterolemia or diabetes was considered if the participants provided a positive answer to the questions: "Did a doctor or a health professional tell you that you have high blood pressure/a high cholesterol level/diabetes?", respectively. Subjects were considered as being treated for hypertension, hypercholesterolemia or diabetes if they answered "daily", "several times per week" or "about once per week" to the questions "During the last seven days, at what frequency did you take medicines for blood pressure/to decrease your cholesterol levels/for diabetes?", respectively. When a participant reported being treated but reported no risk factors, he/she was considered as presenting with the risk factor.

### Other variables

Marital status was grouped into single, married (or cohabitating), divorced and widowed. Education was categorized as follows: (i) no education completed/primary school (referred to as 'basic'); (ii) apprenticeship/secondary level (referred to as 'secondary'); and (iii) tertiary level, which included university and other forms of education after the secondary level (referred to as 'university'). Nationality was defined as Swiss/other. Attendance to a medical consultation within the last 12 months (yes/no) was also analyzed.

### Statistical analysis

Statistical analysis was conducted using SAS v.9.2 (SAS Inc., Cary, NC, USA). As some cantons are very small (for example Appenzell Innerrhoden has only 15,000 inhabitants), it was not possible to directly assess differences between cantons. Hence, it was decided to use regions, as they are administratively defined, aggregate cantons with similar linguistic, geographical or cultural characteristics, and provide an adequate number of participants for analysis. These regions are the same which were used for sampling stratification: West "Leman", West-Central "Mittelland", Northwest, Zurich, North-Eastern, Central and South (Ticino), see Figure 1. The West "Leman" is a French-speaking region, the South (Ticino) an Italian-speaking region, while the other regions are in majority German-speaking.

Quantitative variables were expressed as mean ± standard deviation and qualitative variables as number of participants and (percentage). Bivariate comparisons were performed using analysis of variance (ANOVA) or a chi-square test for quantitative and qualitative variables, respectively.

A first multivariate logistic regression analysis adjusting for gender, age, marital status, educational level and Swiss nationality was performed to compare between regions, using Leman as a reference. For screening, a second analysis with a further adjustment for BMI, smoking status and attendance to medical consultation was also performed. For prevalence, a second analysis with a further adjustment for BMI, smoking status, attendance to medical consultation and screening for the corresponding risk factor was performed. The results were expressed as odds ratio and 95% confidence intervals. Statistically significant differences were considered when p < 0.05.

## Results

### Sample characteristics

Data from 17,879 participants (9862 women, mean age 50.4 ± 17.6 years) was collected. The clinical characteristics of the participants according to the region are summarized in Table 1. Participants living in Ticino were older, while Zurich and Leman had the highest prevalence of highly educated participants. A higher prevalence of divorced participants was also found in these regions, while the lowest prevalence of Swiss nationals was found in Ticino and Leman. Finally, over three quarters of the participants reported having consulted a doctor within the last 12 months, the highest value being found in Leman and the lowest in East Switzerland. Conversely, no differences were found regarding gender distribution between regions (Table 1).

**Table 1:** Main characteristics of the sample, overall and by region, 2007

	Leman	Mittelland	North- West	Zurich	East	Central	Ticino	Switzerland	Test
Sample size	3332	4327	2262	2330	1900	2275	1453	17,879	
Women (%)	1833 (55.0)	2393 (55.3)	1258 (55.6)	1270 (54.5)	1008 (53.1)	1266 (55.7)	834 (57.4)	9862 (55.2)	7.23^NS^
Age (years)	49.9 ± 17.6	50.3 ± 17.7	51.8 ± 17.5	50.8 ± 17.4	49.9 ± 17.6	49.3 ± 17.5	52.9 ± 17.9	50.4 ± 17.6	9.63***
Age^a^	52.1 ± 0.3	51.5 ± 0.3	53.7 ± 0.3	53.9 ± 0.3	52.1 ± 0.4	51.2 ± 0.3	54.3 ± 0.4	NA	18.39***
Educational level									
Basic	518 (15.5)	695 (16.1)	247 (10.9)	196 (8.4)	242 (12.7)	314 (13.8)	259 (17.8)	2471 (13.8)	
Secondary	1815 (54.5)	2548 (58.9)	1382 (61.1)	1310 (56.2)	1188 (62.6)	1395 (61.3)	885 (60.9)	10,523 (58.9)	229.2***
University	999 (30.0)	1084 (25.0)	633 (28.0)	824 (35.4)	470 (24.7)	566 (24.9)	309 (21.3)	4885 (27.3)	
Marital status									
Single	811 (24.3)	1030 (23.8)	562 (24.9)	695 (29.8)	489 (25.8)	584 (25.7)	324 (22.3)	4495 (25.2)	
Married	1767 (53.1)	2309 (53.4)	1188 (52.5)	1071 (46.0)	1045 (55.1)	1258 (55.4)	794 (54.7)	9432 (52.8)	98.23***
Widowed	320 (9.6)	461 (10.7)	240 (10.6)	229 (9.8)	176 (9.3)	216 (9.5)	169 (11.6)	1811 (10.1)	
Divorced	432 (13.0)	525 (12.1)	272 (12.0)	334 (14.4)	187 (9.9)	214 (9.4)	165 (11.4)	2129 (11.9)	
Swiss nationality	2728 (81.9)	3899 (90.1)	1994 (88.2)	2009 (86.2)	1673 (88.1)	2077 (91.3)	1185 (81.6)	15,565 (87.1)	196.1***
Consulted last 12 m	2751 (82.6)	3507 (81.1)	1855 (82.0)	1883 (80.8)	1476 (77.7)	1802 (79.2)	1176 (80.9)	14,450 (80.8)	24.61***

Results are expressed as mean ± standard deviation, as adjusted mean ± standard error or as number of patients and (percentage). **^a ^**adjusted for gender, education, nationality and marital status. NA, not assessable. Statistical analysis by analysis of variance or chi-square test: ^NS^; not significant; *, p < 0.05; **, p < 0.01; ***, p < 0.001

### Obesity and smoking

The prevalences of obesity and smoking according to region are summarized in Table 2. Mean BMI was lower in Zurich and West-Central "Mittelland", and these differences persisted after multivariate adjustment for age, gender, education, nationality and marital status (Table 2). Similarly, prevalence of obesity was lower in Zurich than in West-Central "Mittelland", but no significant between-region differences in obesity levels were found after multivariate adjustment for age, gender, education, nationality and marital status (Table 3).

**Table 2:** Self-reported prevalence and screening the last 12 months of the main cardiovascular risk factors in Switzerland, overall and by region, 2007

	Leman	Mittelland	North- West	Zurich	East	Central	Ticino	Switzerland	Test
Sample size	3332	4327	2262	2330	1900	2275	1453	17,879	
BMI (kg/m^2^)	24.4 ± 4.2	24.7 ± 4.1	24.6 ± 4.0	24.1 ± 4.0	24.5 ± 4.0	24.4 ± 4.0	24.3 ± 4.2	24.5 ± 4.1	6.68***
BMI (kg/m^2^)^a^	24.7 ± 0.1	25.1 ± 0.1	25.0 ± 0.1	24.7 ± 0.1	24.8 ± 0.1	24.8 ± 0.1	24.6 ± 0.1	NA	5.14***
BMI categories (%)									
Normal	2050 (61.5)	2489 (57.5)	1326 (58.6)	1519 (65.2)	1137 (59.8)	1412 (62.1)	893 (61.5)	10,826 (60.5)	
Overweight	1004 (30.2)	1407 (32.5)	718 (31.8)	636 (27.3)	601 (31.7)	674 (29.6)	425 (29.2)	5465 (30.6)	49.46***
Obese	278 (8.3)	431 (10.0)	218 (9.6)	175 (7.5)	162 (8.5)	189 (8.3)	135 (9.3)	1588 (8.9)	
Smoking categories									
Current	918 (27.6)	1148 (26.5)	603 (26.7)	680 (29.2)	551 (29.1)	623 (27.4)	389 (26.8)	4912 (27.5)	
Former	847 (25.4)	1029 (23.8)	563 (24.9)	520 (22.3)	413 (21.8)	479 (21.1)	320 (22.0)	4171 (23.3)	32.17 *
Never	1567 (47.0)	2148 (49.7)	1095 (48.4)	1129 (48.5)	933 (49.2)	1173 (51.6)	744 (51.2)	8789 (49.2)	
Tried to stop smoking	225 (24.5)	282 (24.6)	156 (25.9)	175 (25.7)	160 (29.0)	198 (31.8)	74 (19.0)	1270 (25.9)	25.67***
Hypertension (%)									
Screening	2170 (65.1)	2815 (65.1)	1526 (67.5)	1502 (64.5)	1206 (63.5)	1494 (65.7)	972 (66.9)	11,685 (65.4)	10.10^NS^
Prevalence	843 (25.3)	1194 (27.6)	678 (30.0)	623 (26.7)	497 (26.2)	552 (24.3)	398 (27.4)	4785 (26.8)	24.96***
Treatment^b^	553 (65.6)	777 (65.1)	430 (63.4)	397 (63.7)	285 (57.3)	357 (64.7)	264 (66.3)	3063 (64.0)	12.26 NS
Dyslipidemia (%)									
Screening	1572 (47.2)	1907 (44.1)	969 (42.8)	937 (40.2)	729 (38.4)	862 (37.9)	756 (52.0)	7732 (43.2)	121.8***
Prevalence	730 (21.9)	825 (19.1)	434 (19.2)	447 (19.2)	308 (16.2)	412 (18.1)	333 (22.9)	3489 (19.5)	39.81***
Treatment^b^	344 (47.1)	380 (46.1)	167 (38.5)	166 (37.1)	105 (34.1)	139 (33.7)	159 (47.8)	1460 (41.8)	43.98***
Diabetes (%)									
Screening	1547 (46.4)	1969 (45.5)	1048 (46.3)	1038 (44.6)	818 (43.1)	976 (42.9)	759 (52.2)	8155 (45.6)	39.91***
Prevalence	193 (5.8)	255 (5.9)	137 (6.1)	132 (5.7)	87 (4.6)	92 (4.0)	77 (5.3)	973 (5.4)	15.84 *
Treatment^b^	125 (64.8)	155 (60.8)	69 (50.4)	65 (49.2)	44 (50.6)	53 (57.6)	45 (58.4)	556 (57.1)	13.49 *

Results are expressed as mean ± SD or as number of patients and (percentage). NA, not assessable. Statistical analysis by analysis of variance or chi-square test: ^NS^; not significant; *, p < 0.05; **, p < 0.01; ***, p < 0.001.**^a ^**adjusted for gender, education, nationality and marital status; **^b ^**drug treatment among subjects reporting the risk factor

**Table 3:** Multivariate analysis of the differences between regions in obesity and smoking self-reported prevalence, Switzerland, 2007

	Obesity	Smoking
Lemanic region	1 (ref.)	1 (ref.)
Mittelland	1.20 (1.02 - 1.41)	0.95 (0.86 - 1.06)
Northwest	1.18 (0.98 - 1.43)	1.01 (0.89 - 1.14)
Zurich	0.96 (0.79 - 1.17)	1.10 (0.98 - 1.25)
East	1.02 (0.84 - 1.26)	1.08 (0.94 - 1.22)
Central	1.01 (0.83 - 1.23)	0.99 (0.88 - 1.13)
Ticino	1.02 (0.82 - 1.27)	1.04 (0.90 - 1.20)

Results are expressed as odds ratio and (95% confidence interval). Adjusted for age, gender, marital status, educational level and Swiss citizenship

Slightly more than one quarter of participants smoked, the highest prevalence of smokers being found in Zurich and East Switzerland, while the lowest values were found in West-Central "Mittelland" and North-West regions (Table 2). As for obesity, no significant between-region differences in current smoking levels were found after multivariate adjustment for age, gender, education, nationality and marital status (Table 3). One quarter of current smokers reported having tried to quit within the last 12 months, the percentage being highest in East and Central Switzerland and lowest in Ticino (Table 2). After multivariate adjustment, smokers from East and Central Switzerland had a higher likelihood of reporting they tried to quit: Odds ratio and [95% confidence interval], 1.31 [1.03 - 1.67] and 1.46 [1.16 - 1.83] while no difference was found for Ticino: 0.76 [0.56 - 1.02].

### Hypertension

Almost two thirds of participants reported having their blood pressure levels screened within the last 12 months, and no significant difference was found between regions (Table 2), even after multivariate adjustment for age, gender, marital status, educational level, Swiss citizenship and attending a medical consultation within the last 12 months (Table 4).

**Table 4:** Multivariate analysis of differences between regions for self-reported cardiovascular risk factors, Switzerland, 2007

		Hypertension			Dyslipidemia			Diabetes	
	Screening	Prevalence^a^	Treatment^b^	Screening	Prevalence^a^	Treatment^b^	Screening	Prevalence^a^	Treatment^b^
Leman	1 (ref.)	1 (ref.)	1 (ref.)	1 (ref.)	1 (ref.)	1 (ref.)	1 (ref.)	1 (ref.)	1 (ref.)
Mittelland	1.00 (0.90 - 1.10)	1.10 (0.98 - 1.24)	0.93 (0.75 - 1.15)	0.86 (0.78 - 0.95)	0.83 (0.74 - 0.94)	0.80 (0.64 - 1.01)	0.95 (0.86 - 1.05)	0.98 (0.80 - 1.20)	0.74 (0.48 - 1.13)
Northwest	1.09 (0.96 - 1.23)	1.18 (1.03 - 1.35)	0.86 (0.67 - 1.09)	0.75 (0.67 - 0.85)	0.80 (0.69 - 0.92)	0.59 (0.45 - 0.78)	0.93 (0.83 - 1.05)	0.97 (0.77 - 1.23)	0.54 (0.33 - 0.88)
Zurich	0.97 (0.86 - 1.09)	1.11 (0.96 - 1.27)	0.86 (0.67 - 1.11)	0.72 (0.64 - 0.81)	0.86 (0.74 - 0.99)	0.53 (0.41 - 0.70)	0.93 (0.83 - 1.04)	0.99 (0.78 - 1.26)	0.54 (0.33 - 0.90)
East	1.00 (0.88 - 1.13)	1.07 (0.92 - 1.24)	0.65 (0.50 - 0.85)	0.69 (0.60 - 0.78)	0.71 (0.61 - 0.83)	0.46 (0.34 - 0.63)	0.90 (0.80 - 1.02)	0.78 (0.59 - 1.02)	0.55 (0.31 - 0.97)
Central	1.08 (0.96 - 1.22)	0.98 (0.85 - 1.13)	0.91 (0.70 - 1.18)	0.68 (0.61 - 0.77)	0.87 (0.75 - 1.00)	0.48 (0.36 - 0.63)	0.90 (0.80 - 1.02)	0.70 (0.54 - 0.92)	0.76 (0.44 - 1.33)
Ticino	1.11 (0.97 - 1.27)	0.94 (0.80 - 1.10)	0.89 (0.67 - 1.19)	1.18 (1.03 - 1.36)	0.96 (0.82 - 1.12)	0.87 (0.65 - 1.17)	1.24 (1.09 - 1.42)	0.78 (0.59 - 1.03)	0.71 (0.39 - 1.28)

Results are expressed as odds ratio and (95% confidence interval). Adjusted for age, gender, marital status, educational level, Swiss citizenship, BMI and smoking status and consulted last 12 months; For ^a^, a further adjustment for screening for the risk factor the last 12 months was performed; **^b ^**drug treatment among subjects reporting the risk factor

When asked about their blood pressure status, over one quarter of participants reported being told they had high blood pressure, and significant differences were found between regions, North-West Switzerland presenting the highest levels and Central Switzerland the lowest (Table 2). After multivariate adjustment, a significantly higher likelihood of reporting high blood pressure was found for North-West Switzerland, while no differences were found for the other regions (Table 4).

Almost two thirds of subjects who had been told they had high blood pressure also reported being treated, the highest treatment rates being found for Ticino and the lowest in East Switzerland (Table 2). These findings were partly confirmed after multivariate adjustment, participants from East Switzerland presenting a lower likelihood of being treated, while no differences were found for the other regions (Table 4).

### Dyslipidemia

Slightly over half of the participants reported having their cholesterol levels screened within the last 12 months, with considerable differences between regions. Participants living in Ticino reported the highest screening levels, while participants living in East and Central Switzerland reported the lowest (Table 2). These findings were further confirmed by multivariate adjustment, participants from Ticino presenting a higher likelihood of being screened, while participants from the other regions showed a lower likelihood of being screened (Table 4).

One-fifth of the participants reported being told they had high cholesterol levels, and significant differences were found between regions, the Leman presenting the highest and East Switzerland the lowest rates (Table 2). Multivariate adjustment showed that participants from all regions (except Ticino) had a lower likelihood of reporting being dyslipidemic than participants from Leman (Table 4).

Four out of ten participants who had been told they had high cholesterol levels also reported being treated. Treatment rates were highest in Ticino and Leman and lowest in East and Central Switzerland (Table 2), and these findings were further confirmed by multivariate adjustment (Table 4).

### Diabetes

Less than half of the participants reported having their blood glucose levels screened within the last 12 months, the highest levels being found in Ticino and the lowest in Central and East Switzerland (Table 2). After multivariate adjustment, the participants living in Ticino had a higher likelihood of having their blood glucose assessed, while no differences were found for the other regions (Table 4).

Approximately 5% of all participants reported being told they had diabetes, the highest values being found in Zurich and the lowest in East Switzerland (Table 2). After multivariate adjustment, only participants living in East Switzerland had a lower likelihood of being told they had diabetes, while no differences were found for the other regions (Table 4).

Slightly less than six out of ten participants who had been told they had diabetes reported being treated. The highest treatment rates were found in the Lemanic area and the lowest in Zurich (Table 2). These findings were further confirmed by multivariate analysis, which showed that participants from three regions (Zurich, North-West and East Switzerland) to have a lower likelihood of being treated (Table 4).

## Discussion

To our knowledge, this is the first study to assess the geographical differences in cardiovascular risk factor screening and management in Switzerland. Our results indicate that CVRF screening and management differ between regions and that these differences cannot be accounted for by differences in population age, gender, educational level or migrant status, suggesting that other factors such as local habits might be at stake.

On bivariate analysis, small differences in the prevalence of overweight and obesity were found between regions, but these differences were no longer significant after multivariate adjustment. Overall, our results indicate that excess BMI is evenly distributed throughout Switzerland, although a trend reversal has been shown for Zurich [17]. Hence, it will be of interest to assess regional trends in CVRFs, to assess if there are any differences.

No regional differences were found regarding the reported prevalence of smoking. It is possible that differing smoking policies between cantons within a given region might have reduced the differences between regions but, as reported above, the number of participants for some cantons was too small to draw any valid conclusion. Conversely, current smokers from East and Central Switzerland had a higher likelihood of reporting they tried to quit. Although quitting smoking might be more due to personal motivations than to medical recommendations, our results suggest that some local anti-smoking policies might induce more smokers to try quitting than others. Still, as the prevalence of former smokers in these two regions (East and Central Switzerland) was somewhat lower than in the others, further studies are needed to better assess the actual impact of local policies on smoking prevalence and trends.

No significant differences were found between regions regarding hypertension screening and prevalence. Only two thirds of participants who had been told they were hypertensive reported being treated, a value lower than observed in another Swiss population-based study [18]. Treatment rates were somewhat comparable between regions, with the exception of East Switzerland, which showed significantly lower treatment levels. A possible explanation would be differing socio-economic characteristics of this region as it has been reported in the UK [19], but this hypothesis is rather unlikely as the differences persisted after adjusting for educational level. Another explanation is that health expenditures are lower in the cantons composing this region [11,18], which could lead to lower screening and management efforts; nevertheless, more studies are welcomed to better assess this point. Overall, our data suggest that (i) hypertension screening and management are relatively homogeneous within Swiss regions and (ii) treatment rates could be improved.

Less than half of the participants reported having their cholesterol levels screened the year before, and screening was more frequently reported in French and Italian speaking regions. Similarly, less than half of the participants who had been diagnosed with hypercholesterolemia reported being treated, a finding in agreement with a previous study [20]. Again, most German-speaking regions had significantly lower treatment levels than French or Italian-speaking ones. Overall, our data suggest a clear socio-cultural cleavage regarding screening and management of hypercholesterolemia, practitioners living in French and Italian-speaking regions being more sensitized towards this risk factor. A possible explanation might be the fact that, in Switzerland, the most used equation to assess cardiovascular risk is PROCAM [21], a German-based equation that has not been validated in women neither calibrated for the Swiss population. Conversely, the SCORE equation recommended by the European guidelines [22] which has been calibrated to the Swiss population [23] and shown to present the best cost-effectiveness [24] is seldom applied [21]. Other reasons such as lower health expenditures in the German-speaking regions [11,18] might also intervene, but further studies are needed to better assess the rationale for these regional differences in hypercholesterolemia management and their possible consequences in cardiovascular disease rates. Overall, our results indicate that in Switzerland, (i) the German-speaking regions present lower treatment rates for hypercholesterolemia, and (ii) less than half of the participants diagnosed with hypercholesterolemia actually benefit from drug treatment. It would be of interest to implement the current European guidelines in order to improve the management of hypercholesterolemia in Switzerland.

Less than half of the participants reported having their blood glucose levels screened the year before, this percentage being higher in the South (Ticino). Similarly, less than two thirds of the participants who had been told they had diabetes reported being treated, a value lower than previously reported [25]. As for hypercholesterolemia, most German-speaking regions had significantly lower treatment levels than French or Italian-speaking ones. These findings are partly in agreement with a previous study, which showed significant regional differences in antidiabetic drug prescribing patterns and glycemic control among patients with type 2 diabetes mellitus [14]. Again, the rationale for such regional differences is not clear and might be due to differences in medical expenditures [11], or to differing practices. Overall, our data indicate that (i) management of diabetes varies according to region in Switzerland, the German-speaking regions presenting lower treatment rates, and (ii) less than two thirds of the participants diagnosed with diabetes actually benefit from drug treatment. Again, it would be of interest to apply the current guidelines on screening and management of diabetes [26-29] to optimize outcome and health expenditures.

The between-regional differences observed regarding CVD prevention might partly be due to differing local health policies or to differing health insurance systems. For instance, for most benefits covered by health insurance, tariffs are set at national level for medical goods or negotiated at cantonal level for services [30], leading to different insurance premiums between cantons and/or regions. Further, one third of the Swiss population also contracted a private supplementary health insurance, and over a thousand different supplementary health insurance products existed in 2011 [30]. Finally, a recent OECD report [30] concluded that the split governance between cantons and the federal level leading to a lack of political leadership and drive for reforms in the Swiss health system; the report also concluded that Switzerland should overcome co-ordination problems to define national policies for prevention and health promotion.

This study has some limitations. First, most data were self-reported, which could lead to an underestimation of the true prevalence of the main cardiovascular risk factors, as it has been shown that a significant percentage of the population is not aware of their status [8,18,20,25]. Conversely, it is possible that more healthy-conscious subjects participated, which would increase screening and treatment rates. Nevertheless, this would not impact between-region differences and would suggest that true screening and treatment rates are actually lower than the ones presented, further increasing the urge of implementation procedures. The main strengths of this study is that it can be considered as representative of the Swiss population, it allowed to assess not only prevalence but also screening and management of the main CVRFs, and to adjust for a variety of co-factors, including educational level and medical consultations. It is also possible that some participants reporting high levels of cardiovascular risk factors might not justify being treated as their overall cardiovascular risk, as assessed by the common risk equations, might be below the treatment threshold. Still, it has been shown that non-calibrated CVD risk equations overestimate risk among Swiss [31], which would prompt Swiss GPs to treat their patients more frequently than actually needed. Further, a recent survey conducted in 66 general practices in 12 European countries showed that blood pressure, lipid and glucose control are completely inadequate with most patients not achieving the targets defined in the prevention guidelines [32]. Hence, it is likely that considerable progress can still be achieved regarding CVD prevention in Switzerland. Finally, although data obtained from Health Interview Surveys parallel those obtained using examination surveys [33], the best option would be to associate the results from a Health Interview and a Health Examination Survey, as the former allow the collection of objectively measured data. Furthermore, the implementation of a standardized Health Examination Surveys, based in questionnaires and measurements, would allow a better comparison between countries [34-36]. Still, the implementation of such a study is costly, usually leading to a smaller sample size (with possible biases regarding minorities or specific population groups), and despite recommendations [34] several European countries (Austria, Belgium, Portugal...) never conducted such a survey. In Switzerland, the ongoing 2012 National Health Survey follows a methodology similar to the previous ones^2 ^and the results will be compared to local or regional surveys based on objectively measured data.

## Conclusion

In Switzerland, cardiovascular risk factor screening and management differ between regions and these differences cannot be accounted for by differences in populations' characteristics. The impact of these differing health strategies on cardiovascular disease incidence and mortality should be further assessed.

## Endnotes

^1^http://www.bfs.admin.ch/bfs/portal/fr/index/themen/16/04/key/approche_globale.indicator.30101.301.html?open=2,1#1 (in French)

^2^http://www.bfs.admin.ch/bfs/portal/fr/index/infothek/erhebungen__quellen/blank/blank/ess/01.html, assessed 7 February 2012

## Competing interests

The authors declare that they have no competing interests.

## Authors' contributions

PMV made the statistical analysis and wrote part of the article; FP revised the article for important intellectual content. PMV had full access to the data and is the guarantor of the study. All authors read and approved the final manuscript.

## Pre-publication history

The pre-publication history for this paper can be accessed here:

http://www.biomedcentral.com/1471-2458/12/246/prepub
